# Genome-Wide Analysis of Polygalacturonase Gene Family Reveals Its Role in Strawberry Softening

**DOI:** 10.3390/plants13131838

**Published:** 2024-07-04

**Authors:** Mantong Zhao, Ruixin Hu, Yuanxiu Lin, Yeqiao Yang, Qing Chen, Mengyao Li, Yong Zhang, Yunting Zhang, Yan Wang, Wen He, Xiaorong Wang, Haoru Tang, Ya Luo

**Affiliations:** College of Horticulture, Sichuan Agricultural University, Chengdu 625014, China; 18080011314@163.com (M.Z.); 19383028396@163.com (R.H.); linyx@sicau.edu.cn (Y.L.); 17828812043@163.com (Y.Y.); supnovel@sicau.edu.cn (Q.C.); limy@sicau.edu.cn (M.L.); zhyong@sicau.edu.cn (Y.Z.); asyunting@sicau.edu.cn (Y.Z.); wangyanwxy@sicau.edu.cn (Y.W.); hewen0724@sicau.edu.cn (W.H.); wangxr@sicau.edu.cn (X.W.); htang@sicau.edu.cn (H.T.)

**Keywords:** polygalacturonase, strawberry, fruit softening, fruit firmness

## Abstract

Fruit softening is a prominent attribute governing both longevity on shelves and commercial worth. Polygalacturonase (PG) plays a major role in strawberry fruit softening. However, the *PG* gene family in strawberry has not been comprehensively analyzed. In this study, 75 *FaPG* genes were identified in the octoploid strawberry genome, which were classified into three groups according to phylogenetic analysis. Subcellular localization prediction indicated that *FaPGs* are mostly localized to the plasma membrane, cytoplasm, and chloroplasts. Moreover, the expression of *FaPGs* during strawberry development and ripening of ‘Benihoppe’ and its softer mutant was estimated. The results showed that among all 75 *FaPGs*, most genes exhibited low expression across developmental stages, while two group c members (FxaC_21g15770 and FxaC_20g05360) and one group b member, FxaC_19g05040, displayed relatively higher and gradual increases in their expression trends during strawberry ripening and softening. FxaC_21g15770 was selected for subsequent silencing to validate its role in strawberry softening due to the fact that it exhibited the highest and most changed expression level across different developmental stages in ‘Benihoppe’ and its mutant. Silencing FxaC_21g15770 could significantly improve strawberry fruit firmness without affecting fruit color, soluble solids, cellulose, and hemicellulose. Conversely, silencing FxaC_21g15770 could significantly suppress the expression of other genes related to pectin degradation such as *FaPG-like*, *FaPL*, *FaPME*, *FaCX*, *FaCel*, *FaGlu*, *FaXET,* and *FaEG*. These findings provide basic information on the *FaPG* gene family for further functional research and indicate that FxaC_21g15770 plays a vital role in strawberry fruit softening.

## 1. Introduction

Strawberry (*Fragaria* × *ananassa* Duch.) serves as a prominent model plant for investigating non-respiratory jump fruits due to its vibrant color, distinctive flavor, delightful sweet and sour taste, and abundant content of anthocyanins, amino acids, and other essential nutrients. Consequently, it has gained significant popularity among consumers [1]. However, the edible fruits are derived from the receptacle and lack a protective peel covering. As they reach maturity, these fruits gradually soften and become highly susceptible to damage, thereby significantly compromising their overall quality and economic value [2,3]. Therefore, the enhancement in fruit firmness and preservation of fruit quality have become prominent areas of investigation in current research.

Fruit softening, a prominent attribute of maturation in the majority of succulent fruits, plays a pivotal role in governing both longevity on shelves and commercial worth. Softening is primarily caused by the degradation of cell wall components such as cellulose, hemicellulose, and pectin, leading to alterations in cell wall structure and intercellular adhesion. The decomposition of pectin plays a pivotal role in fleshy fruit softening [4]. Key genes involved in regulating pectin degradation in fleshy fruits include pectin methylesterase (*PME*), pectate lyase (*PL*), and polygalacturonase (*PG*) [5]. Following PME-catalyzed demethylation, PL further breaks down pectin into unsaturated galacturonic acid, while PG hydrolyzes α-1,4-galacturonic acid into glutaric acid.

PG is one of the most studied pectinases involved in fruit softening. It has been suggested that various fruits including tomato, avocado, and peach show high PG activity, which is correlated with the rate of softening. Some other fruits such as melon, apple, and strawberry exhibit very low PG activity. To date, the efficacy of *PG* in promoting fruit softening and reducing firmness has been convincingly demonstrated in the ripening processes of multiple fruits, including tomatoes (*SlPG49*) [6], apples (*MdPG1*) [7], and fig fruit (*FcPG12*) [8]. In woodland strawberry, a total of 82 *PG* genes have been identified, and their expression profiles have been analyzed in the ripe receptacle [9]. In cultivated strawberry, two *PG* genes have been characterized, namely *FaPG1* (NCBI accession no. AF380299) and *FaPG2* (NCBI accession no. AY280662) [10,11,12]. Both genes were found to be up-regulated during the fruit ripening process and showed a negative correlation with fruit firmness. Additionally, transgenic strawberry plants with *FaPG1* suppression and knock out were previously successfully generated through antisense transformation or CRISPR/Cas9 genome editing techniques [9,11,13,14]. Remarkably, the fruit firmness was significantly enhanced without any noticeable impact on other ripening-associated attributes such as color, weight, or soluble solids content. Regarding *FaPG2*, although functional analysis has not been addressed, it has been observed that the active site of the deduced protein differed from that found in known *PGs* [12]. Moreover, *FaPG2* exhibited a higher expression level in ripe fruit from soft cultivars compared to those from firm genotypes [12]. Furthermore, a comprehensive RNA sequencing (RNAseq) study conducted on the firm cultivar ‘Camarosa’ detected expression of *FaPG1*, but did not detect expression of FaPG2 [15]. However, a comprehensive genome-wide analysis of the PG family in cultivated strawberries is still missing.

The strawberry ‘Benihoppe’ currently holds the distinction of being the largest cultivated variety in China. In our previous research endeavors, our team successfully identified a stable mutant strain of ‘Benihoppe’ strawberries that exhibited notable characteristics including dwarf plants, deep red fruits, smaller fruit size, and reduced fruit firmness when grown in the field. Our earlier investigations have already elucidated the underlying cause behind this mutant’s color change [16]. Interestingly, we have also discovered differential expression of a *PG* gene in both ‘Benihoppe’ and its mutant counterpart. Consequently, we propose that this gene serves as a pivotal factor contributing to the softening phenomenon observed in the mutant fruits. Due to the limited availability of fundamental information regarding strawberry *PG* genes, this study primarily focused on conducting a genome-wide identification and analysis of the *PG* gene through genetic mapping, gene structure analysis, and evolutionary analysis. Furthermore, we compared the transcription abundance of *FaPG* family members during different stages of fruit development in two distinct strawberry materials, and subsequently selected a most differentially expressed gene (FxaC_21g15770) for further functional validation. The results presented in this study provide a foundation for further understanding the role of *PG* family members in regulating the strawberry fruit softening process, while also offering potential candidate genes for breeding strawberries with higher firmness.

## 2. Materials and Methods

### 2.1. Plant Materials

The plants of the strawberry cultivar ‘Benihoppe’ and its mutant were grown in a greenhouse located in Hanyuan, Ya’an, China. The mutant was a natural mutant identified during the cultivation of ‘Benihoppe’ strawberries. In this experiment, the fruits of the ‘Benihoppe’ strawberry and its mutant (MT) were used as materials. According to our previous study [16], they were characterized based on their developmental stages: large green (LG), partial red (PR), full red (FR), large green (LG), and partial red (MT). The fruits were collected between January and February 2023 from a strawberry planting base in Hanyuan County, Sichuan Province. Immediately after harvest, the fruit firmness was determined, and the samples were frozen at −80 °C for further analysis. Each set of biological replicates consisted of 10 fruits, with a total of three replicates conducted.

### 2.2. Identification, Phylogeny and Characterization of PG Gene Family in Strawberry Genome

The genome and protein sequence data of *Fragaria vesca*, *Fragaria* × *ananassa*, and *Arabidopsis thaliana* were obtained from the Genome Database for Rosaceae (GDR) (https://www.rosaceae.org/) and The Arabidopsis Information Resource (TAIR) database (https://www.arabidopsis.org/). The *FaPG* protein was identified using a hidden Markov model of the Polygalacturonase (*PG*) domain (PF00295) from the Pfam database (http://pfam.xfam.org/). The presence of *PG* domains in all candidate *PGs* was verified using a Hidden Markov Model search with default parameters retrieval against the Conserved Domain Database (CDD) at NCBI (http://www.ncbi.nlm.nih.gov/cdd/) and the SMART database at EMBL-EBI (https://smart.embl-heidelberjg.de/). The ProtParam tool, available on the ExPASy server (http://www.expasy.org/tools/protparam.html), was used to predict gene ID, amino acid length, open reading frame size, protein molecular weight, theoretical isoelectric point, instability index, aliphatic index, grand average of hydropathicity score (GRAVY), chromosome number, chromosome location, and subcellular localization. Coding sequences of less than 100 amino acids were discarded. TBtools software (v. 2.003) was utilized to visualize the chromosomal distribution diagram of the *FaPG* gene family. MEGA 11.0 software (v. 11.0.13) was used to implement the neighbor-joining method for constructing the phylogenetic tree, while the Evolview site (http://120.202.110.254:8220/evolview)was employed for beautifying the evolutionary tree visualization. The MEME suite (https://meme-suite.org/meme/) was used to annotate conserved domains and motifs. WOLF PSORT (https://www.genscript.com/wolf-psort.html/) was used to predict subcellular localization. TBtools was employed to visualize conserved motifs, conserved domains, and gene structure within FaPGs family members. The Circos software was used to render *FaPGs* gene collinearity analysis (http://circos.ca/software/download/), while MCScanX software was employed to identify homologous genes in the *Fragaria* × *ananassa*, *Fragaria vesca*, and *Arabidopsis thaliana FaPG* gene families (http:/chibba.pgmluga.edu/mcscan2).

### 2.3. RNA Extraction, cDNA Synthesis and PCR Amplification

Total RNA was extracted from three developmental stages (LG, PR, and FR) of ‘red’ and ‘MT’ fruits using an improved CTAB (cetyltrimethyl ammonium bromide) method [17]. The first strand of cDNA was synthesized following the TransScript^®^ All-in-One first-strand cDNA Synthesis Super Mix for PCR user manual provided by Transgen Biotechnology Co., Ltd. (Beijing, China). A 20 µL reaction system was employed, consisting of a 4 µL reverse transcription kit (5× TransScript^®^ All-in-one SuperMix), 6 µL RNA, and 10 µL RNase-free water. Incubation at 42 °C for 15–20 min followed by heating at 85 °C for 5 min were performed. The results of PCR amplification were assessed through electrophoresis on a 1.0% agarose gel.

### 2.4. RNA Sequencing and Transcriptome Analysis

Total RNA was extracted from three developmental stages of ‘Benihoppe’ and ‘MT’ fruit, followed by the construction of RNAseq libraries using the Illumina Next^®^UltraTM RNA Library Preparation Kit (NEB, USA) according to the standard protocol. Novogene (Beijing, China) was used to perform library clustering and sequencing on the Hiseq-2500 (Beijing, China) platform (150 bp paired-end reads), generating a total of 18 libraries with three replicates per treatment. FASTQ reads derived from CASAVA base calling were screened for low-quality (Q < 20) bases and adaptors by utilization of trim-galore (v0.6.6). The cleaned reads were mapped onto the strawberry genome and quantified using the accurate Fanse3 mapping pipeline [18] with the parameters -L160, -E5, -S14, and -B. The genome data were obtained from the GDR database (https://www.rosaceae.org/species/fragaria_×_ananassa/genome_v1.0.a1) [19]. The edgeR (v3.34.1) R package [20] was employed to detect the differentially expressed transcripts with the raw reads count using the exact negative binomial test. Only those transcripts with significant changes (log^2^ transformed fold change (log^2^FC) greater than 0.5 or less than −0.5) and an adjusted *p*-value of ≤0.05 were considered as differentially expressed. The KEGG pathway and GO enrichment analysis were conducted using the R package cluster Profiler (v4.2.0) [21].

### 2.5. Vector Construction and Instantaneous Silencing

The fragment sequence of FxaC_21g15770 was amplified using specific primers, while the *FaPG1* fragment 2 sequence was amplified using RNAi-FaPG1-C and RNAi-*FaPG1*-D primers (Supplement Table S1). The P2301RNAi vector available in the laboratory was employed for vector construction. A specific 300 bp target gene fragment was inserted on one side of the PDK insert fragment, with a reverse complementary fragment of the target gene inserted on the other side to achieve silencing of the target gene. The P2301 RNAi expression vector served as a control. During the de-greening stage, strawberry fruit were temporarily silenced using the agrobacterium-mediated method with a P2301 RNAi *FaPG1* construct. After dark cultivation for 5 days, fruit hardness and color difference were assessed before freezing at −80 °C for further analysis. Each replicate consisted of 10 fruits, and the experiment was repeated three times.

### 2.6. Fruit Color, Hardness, and Soluble Solid Content Determination

Fruit skin color indexes (L*, a*, b*) were determined by a color difference meter (Mineng, Osaka, Japan). Soluble solids were measured using a digital display sugar meter. The hardness of strawberries was assessed with a GY-4 fruit hardness tester. Each replicate consisted of 10 fruits, and the experiment was repeated three times.

### 2.7. Determination of Pectin, Cellulose and Hemicellulose Content

The pectin content in strawberry fruit was quantified using carbazole colorimetry [22]. A 1.0 g strawberry sample was weighed and ground in a mortar, transferred to a 50 mL calibrated centrifuge tube, and mixed with 25 mL of 95% ethanol. The mixture was then boiled for 30 min, cooled to room temperature, and centrifuged at 8000 r/min for 15 min. The supernatant was discarded. Subsequently, the residue was treated with additional portions of boiling ethanol (95%) three to five times. To dissolve the pectin, the residue was mixed with 20 mL of distilled water and kept in a water bath at 50 °C for 50 min. After cooling to room temperature again, it underwent another round of centrifugation at 8000 r/min for 15 min. The resulting supernatant was transferred into a volumetric bottle (10 mL) and its absorption value at a wavelength of 530 nm was measured three times to determine the soluble pectin content as a mass fraction. The hydrolysis of protopectin began with heating in a boiling water bath for one hour followed by centrifugation to obtain the supernatant, of which the volume was adjusted to be fixed at 100 mL before measuring its absorption value at a wavelength of 530 nm. The measurement process was repeated three times, and the original pectin content was expressed as a mass fraction. 

The cellulose (CLL) content kit and hemicellulose content kit provided by Suzhou Gris Biotechnology Co., Ltd. (Suzhou, China) were employed for the determination of cellulose and hemicellulose content in fruit samples. A 0.02 g sample was taken and subjected to a water bath at 50 °C for 20 min, followed by centrifugation to obtain the precipitate. Subsequently, 1.5 mL of 80% ethanol was added for agitation and mixing, repeating the aforementioned steps as necessary. Afterwards, an extraction solution of 1 mL was added to a water bath at 90 °C for 15 min, followed by centrifugation to once again collect the precipitate. Then, 1 mL of acetone was added for agitation and mixing before another round of centrifugation to collect the precipitate. The obtained precipitate was dried through incubation at 90 °C for a duration of 20 min. Following this step, reagent 1 (0.2 mL) was added and incubated at a temperature of 110 °C for one hour before it was cooled and subjected to room temperature centrifugation lasting five minutes. Finally, the supernatant was collected for measurement purposes. Cellulose and hemicellulose contents were determined separately by subjecting them, respectively, to a boiling water bath treatment with mixed reagents in order to measure their absorbance levels at wavelengths of 620 nm and 460 nm, respectively. The experimental procedure described above was repeated three times.

### 2.8. Measurement of PG Enzyme Activity

The determination of PG enzyme activity was performed according to the method described previously [23]. Briefly, 1 g of sample was extracted with 95% ethanol for 10 min and then centrifuged at 4 °C for 20 min. The supernatant was discarded, and the pellet was further extracted with pre-cooled 80% ethanol. After another round of centrifugation, a total of 5 mL pre-cooled enzyme extract was added to the precipitate, followed by centrifugation at 4 °C. The resulting supernatant was collected. This process was repeated three times. Subsequently, two 50 mL centrifuge tubes were taken, and each tube received (a) 1 mL of acetate-sodium acetate buffer solution (pH5.5) and (b) 0.5 mL of polygalacturonic acid solution (10″).

### 2.9. qRT-PCR Analysis

RT-qPCR-based expression analysis was performed using SYBR Green Premix Ex TaqTM (Takara, Tokyo, Japan) on a CFX96 RT-qPCR system (Bio-Rad, Hercules, CA, USA). The total RNA was extracted from the plant sample utilizing the improved cetyltrimethylammonium bromide (CTAB) method. Strawberry tissues and fruit at various developmental stages were collected as described in a previous study. The first strand cDNA synthesis followed the operating manual of the PrimeScript^TM^ RT reagent Kit with a gDNA Eraser (Takara, Tokyo, Japan). Relative expression levels were determined using the 2^−ΔΔCt^ method [24], with the 26-18S interspacer RNA sequence [25] serving as an internal reference. Expression data are presented as mean ± standard deviation (SD) of three independent biological replicates. Specific primers for RT-qPCR were designed using NCBI online tools. Supplementary Table S3 provides a list of all primer sequences used.

### 2.10. Statistical Analysis

The data were processed using Excel 2010 software and SPSS 20.0 for statistical analysis. The LSD minimum significant difference method was employed to compare the significance of differences. Results are presented as mean ± standard deviation. A *p*-value ≤ 0.05 is indicative of a statistically-significant distinction between both samples, indicated with an asterisk (*). A *p*-value ≤ 0.01 indicates extreme significance, marked with double asterisks (**). The aforementioned experiments were conducted thrice.

## 3. Results

### 3.1. Identification of FaPGs Family in Strawberry

**Figure 1:**
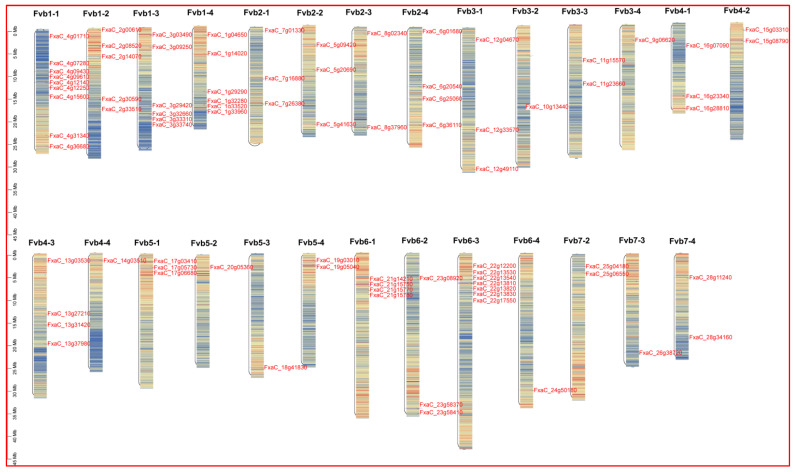
Location and distribution of *FaPGs* on chromosomes. The Fvb7-1chromosome, without any *FaPGs* gene localization, is not shown.

After conducting homologous alignment and verifying conserved domains, we successfully identified a total of 75 genes encoding *FaPGs* proteins (Table S3). The *FaPGs* proteins consist of 92 amino acids, with a range between 92–1513 amino acids (aa). Notably, the majority of *FaPGs* proteins (51) are concentrated within the range of 300–500 aa (Table S3). The ORF size for FaPGs ranges from 276 bp (FxaC_16g23340) to 45,395 bp (FxaC_22g13830). Furthermore, the molecular weight varies widely from 10.2 kDa to 16.1 kDa, while the pI ranges from 4.92 to 9.64. All proteins exhibit an instability index ranging from 23.08 to 53.41, along with an aliphatic index spanning from 69.89 to 104.96, and hydrophilicity ranging from −0.429 to 0.33. Subcellular localization predictions indicate that most *FaPG* proteins are located in the plasma membrane, cytoplasm, and chloroplasts; however, only a few of them are found in the nucleus, mitochondria, or cell wall. Moreover, chromosomal locations reveal that these 75 *FaPG* genes are widely distributed across 27 of the 28 chromosomes in the cultivated strawberries (Figure 1), with the gene number varying from 1 to 9 (Figure 1). It is important to note that the distribution of the *FaPG* is different on each chromosome, and it is more concentrated on Fvb1-1, Fvb1-3, Fvb1-4, Fvb2-4, Fvb4-3, Fvb6-l, and Fvb6-3. Fvbl-l contains the highest number of *FaPG* genes, namely 9, in contrast to Fvb7-l, which lacks any *FaPG* genes.

### 3.2. Phylogenetic Analysis

**Figure 2:**
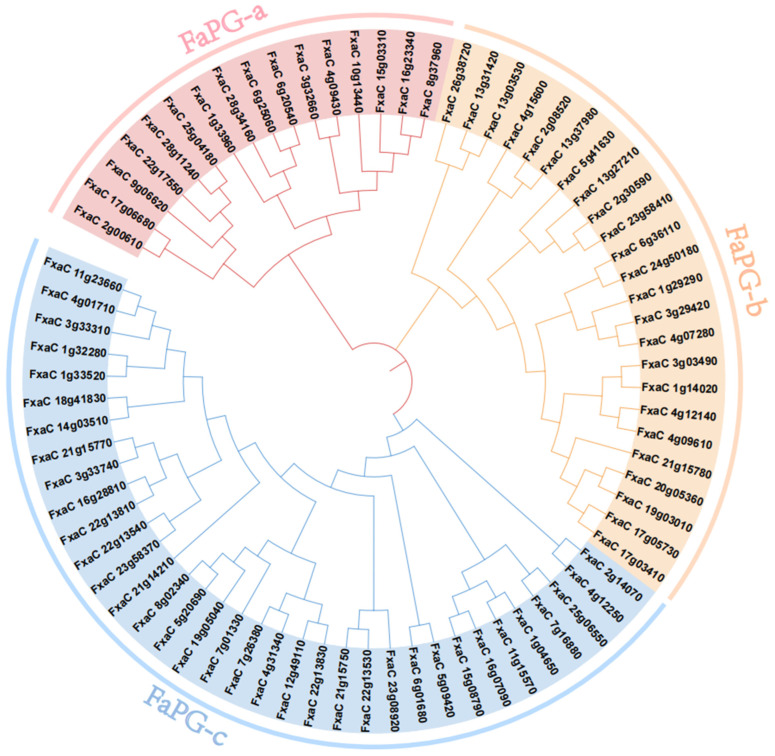
Phylogenetic analysis of *FaPGs* proteins in strawberry (*Fragaria × ananassa*). The different colors show the different subfamilies.

To investigate the evolutionary relationships among *FaPG* proteins across the strawberry genome, a phylogenetic tree was constructed using 75 strawberry *FaPG* protein sequences (Figure 2). Based on the branching pattern, *FaPGs* can be classified into three subfamilies: *PG*-a, *PG*-b, and *PG*-c. Among these subfamilies, *PG*-c has the highest number of members with 35 family members, accounting for 47% of the total. On the other hand, *PG*-a has the fewest family members with only 16, while *PG*-b has 24 members.

### 3.3. Structure and Motif Localization Analysis of FaPGs Genes

**Figure 3:**
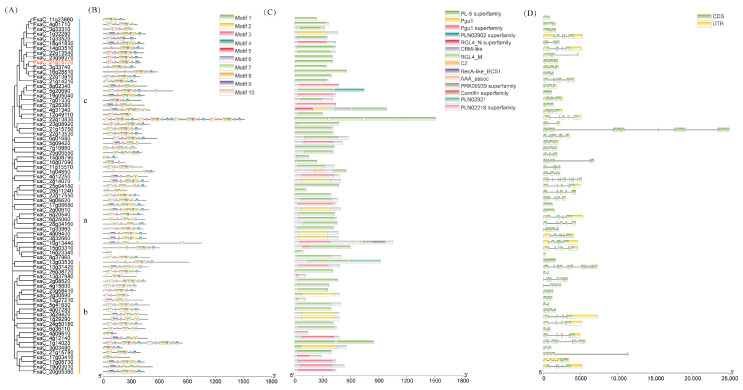
Phylogenetic relationships, conserved protein motifs, and gene structure analysis of *FaPGs*. (**A**) Phylogenetic tree of *FaPGs* gene family (Based on the branching pattern, *FaPGs* can be classified into three subfamilies: *PG*-a, *PG*-b, and *PG*-c.); (**B**) distribution of *FaPGs* protein motifs; (**C**) conserved domain in *FaPGs* proteins; (**D**) exon-intron structure of *FaPGs* genes.

In order to further elucidate the structural characteristics of the *FaPGs* genes, we conducted a comprehensive analysis of the gene structure of *FaPGs* (Figure 3). As in Figure 2, a phylogenetic tree of *FaPGs* is shown in Figure 3A. A total of 10 distinct motifs were identified in the sequence of *FaPGs* (Figure 3B). With the exception of FxaC_3g33310, FxaC_1g33520, FxaC_4g09610, and FxaC_16g07090, nearly all members of the *FaPGs* exhibit Motif 2, indicating a remarkable level of conservation among these genes. However, it is noteworthy that FxaC_16g23340 only contains two conserved motifs, namely motif 2 and motif 6. Similarly, both FxaC_15g08790 and FxaC_4g09610 possess three conserved motifs each: motif 2, motif 4, and motif 6; and motif 1, motif 5, and motif 8, respectively. Conserved domain analysis revealed that the majority of the *FaPG* members harbor PL-6 superfamily domains along with Pgu1 and Pgu1 superfamily domains. Additionally, group a member FxaC_15g03310 also exhibits the Cornifin superfamily domain, while FxaC_10g13440 possesses the RecA-like_BCS1, AAA_assoc, and PRK08939 superfamily domains. Amongst them all, FxaC_4g31340 is characterized by the RGL4_N superfamily, CBM-like, and RGL4_M domains (Figure 3C). Meanwhile, the exon-intron structure analysis of 75 *FaPG* genes revealed that three *FaPGs* in groups a and c had no introns, while the other members showed discontinuous sequences due to the distribution of intron numbers. The results show that the number of exons of *FaPGs* members ranges from 1 to 18, with FxaC_21g15750 having the maximum number of 18 exons.

### 3.4. Synteny Analysis

**Figure 4:**
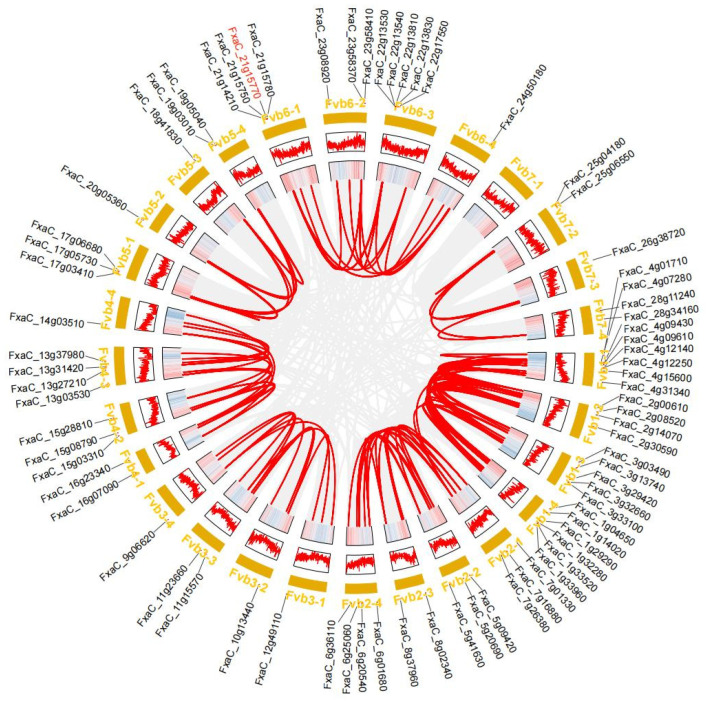
Segmental repetition and collinearity analysis of *FaPGs*. The 75 *FaPG* genes are labeled with short lines on the chromosome, and the red curve indicates collinear gene pairs.

**Figure 5:**
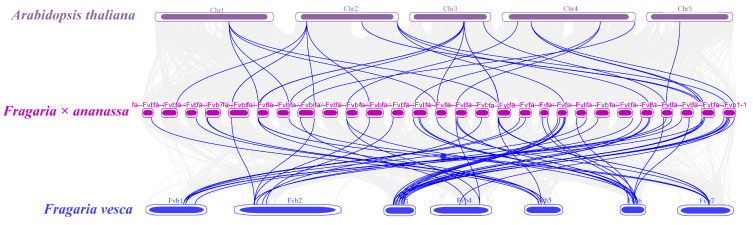
Collinearity analysis of *PG* genes in *Arabidopsis*, cultivated strawberry, and woodland strawberry genomes. The gray lines represent collinear blocks within the three genomes, while the blue lines represent collinear PG gene pairs. Dark purple, light purple, and blue round rectangles represent the chromosomes of the genomes of *Arabidopsis thaliana*, cultivated strawberry, and woodland strawberry, respectively. The chromosome number is shown on one side of the chromosome.

To elucidate the evolutionary relationship of *FaPG* genes, collinearity analysis was conducted among the genomes of *Arabidopsis thaliana*, *Fragaria vesca* (woodland strawberry), and *Fragaria* × *ananassa* (cultivated strawberry). A total of 118 *FaPG* gene pairs were identified in the collinearity analysis of *Fragaria* × *ananassa* (Figure 4), with most of them being fragment duplications located on the same or adjacent chromosomes. Comparing genomic homology, there are a total of 45,171 homologous blocks between *Arabidopsis thaliana* and *Fragaria* × *ananassa* genomes. Additionally, *Fragaria* × *ananassa* and *Fragaria vesca* exhibit 71,819 homologous blocks (Figure 5). These findings indicate that *Fragaria* × *ananassa* is closely related to *Fragaria vesca*.

### 3.5. Changes in the Firmness of ‘Benihoppe’ and Its Mutant during Fruit Development

**Figure 6:**
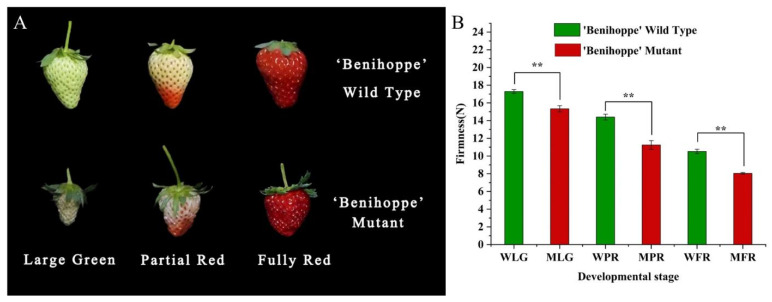
Differences in phenotype and hardness of ‘Benihoppe’ and its mutants during fruit development. (**A**) Fruit appearance of ‘Benihoppe’ and its mutants; (**B**) fruit firmness of ‘Benihoppe’ and its mutants. WLG, WPR and WFR indicate the large green, partial red, and fully red developmental stages of ‘Benihoppe’ separately; MLG, MPR, and MFR represent the large green, partial red, and fully red developmental stages of mutant individually. double asterisks represent significant differences between the two samples at *p* ≤ 0.01 level. Below is the same.

There were significant phenotypic differences in fruit appearance between ‘Benihoppe’ and its mutant (Figure 6A). The ‘Benihoppe’ fruit exhibited larger size and smaller sepals compared to the mutant throughout development, with a bright red color at maturity, while the mutant fruit appeared dark red. Furthermore, measurements were conducted on the firmness of both materials at three stages of fruit development, indicating that the firmness of ‘Benihoppe’ was significantly higher than that of MT at all three stages. At the fully ripe stage, the firmness values for ‘Benihoppe’ and mutant fruits were recorded as 17.39 N and 15.03 N, respectively (Figure 6B), suggesting a 1.3 times higher of firmness in ‘Benihoppe’ compared to the mutant. These findings indicate that besides visual characteristics, the hardness of the mutant fruit has also undergone genetic changes.

### 3.6. Transcriptional Differences of FaPGs Involved in Fruit Development and Ripening of ‘Benihoppe’ and Its Mutant

**Figure 7:**
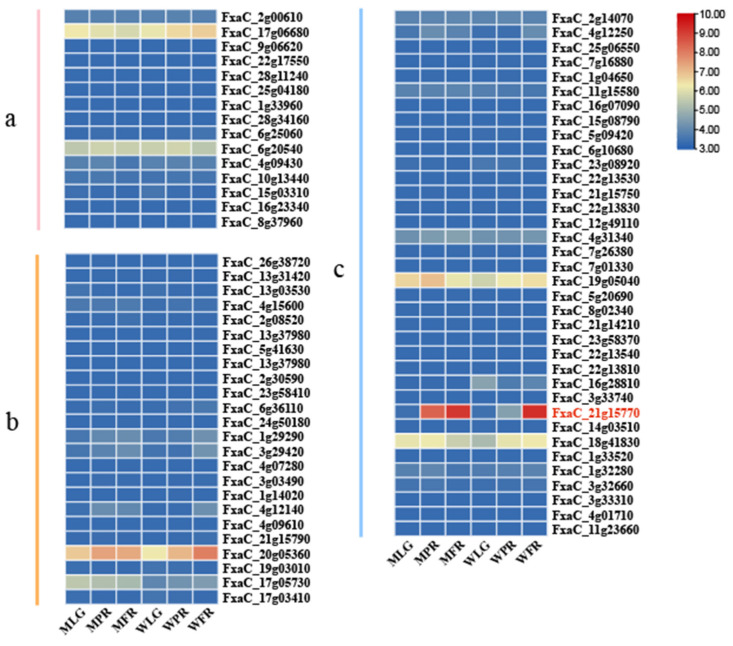
Transcriptional abundances of *FaPGs* genes at different fruit development and ripening stages of ‘Benihoppe’ and its mutants. The FPKM values of *FaPGs* genes were transformed by log10 (FPKM + 1), and the heat map was constructed by TBtools software. Based on the branching pattern, *FaPGs* can be classified into three subfamilies: *PG*-a, *PG*-b, and *PG*-c. WLG, WPR, and WFR indicate the large green, partial red, and fully red developmental stages of ‘Benihoppe’ separately; MLG, MPR, and MFR represent the large green, partial red, and fully red developmental stages of the mutant individually.

Transcriptional differences of *FaPGs* were analyzed using transcriptome data to identify members associated with strawberry fruit ripening and softening. Figure 7 illustrates that group b and group c exhibited higher expression at the LG, PR, and FR stages of fruit development than group a, indicating similarity in gene expression among homologous gene pairs. The expression levels of FxaC_21g15770, FxaC_20g05360, and FxaC_19g05040 were lower during the LG stage but gradually increased with fruit maturation in both ‘Benihoppe’ fruits and their mutants. Furthermore, these genes demonstrated higher expression levels during the PR and FR stages of mutants with reduced hardness compared to ‘Benihoppe’. These findings suggest that the group c members, including FxaC_21g15770 and FxaC_20g05360, and the group b member FxaC _19g05040, may be related to strawberry fruit ripening and softening. Notably, among these three genes, the expression of FxaC_21g15770 changed the most. Compared to ‘Benihoppe’, this gene showed a 245% increase in expression during the PR stage and a 12% increase during the FR stage in mutant fruit. Therefore, FxaC_21g15770 was selected for subsequent function validation in strawberry fruit ripening and softening.

### 3.7. Silencing of FxaC_21g15770 Significantly Improved Fruit Firmness

**Figure 8:**
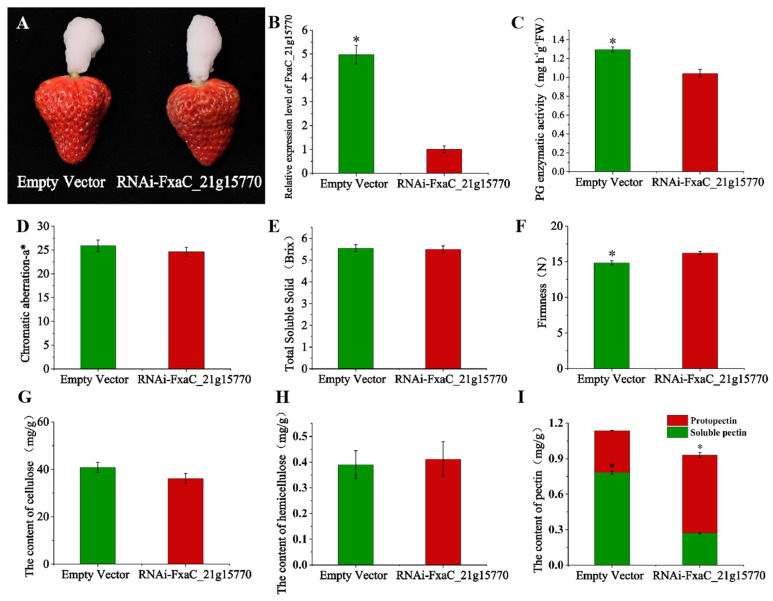
Instantaneous silencing of *FaPG1* in strawberry fruit. (**A**) Phenotype of strawberry infiltrated with empty vector (control) and silenced *FaPG1* recombinant plasmid; (**B**) relative expression of *FaPG1* in *FaPG1* silenced fruit and control fruit; (**C**) PG enzyme activity of *FaPG1* silenced fruit and control fruit; (**D**) a* value of color in *FaPG1* silenced fruit and control fruit; (**E**) soluble solids content of *FaPG1* silenced fruits and control fruits; (**F**) hardness of *FaPG1* silenced fruits and control fruits; (**G**) cellulose content in no-load (control) and silenced *FaPG1*, hemicellulose content in *FaPG1* silenced fruits and control fruits, and pectin content; (**H**) the content of hemicellulose of *FaPG1* silenced fruits and control fruits; (**I**) *FaPG1* silenced fruits and control fruits. RNAi, RNA interference. Single asterisk represent significant differences between the two samples at *p* ≤ 0.05 level.

In order to further investigate the role of FxaC_21g15770 in regulating strawberry fruit ripening, we transiently suppressed the expression of FxaC_21g15770 in strawberry fruit (Figure 8A). The results demonstrated a fivefold reduction in FxaC_21g15770 expression compared to the control group, indicating successful suppression (Figure 8B). The detection of PG enzyme activity revealed a significantly lower level in FxaC_21g15770-silenced fruits at 1.04 mg·h^−1^ g^−1^ FW compared to the control group’s value of 1.30 mg·h^−1^ g^−1^ FW (Figure 8C). Moreover, suppression of FxaC_21g15770 did not affect the a* value related to strawberry fruit coloration, or soluble solid content (Figure 8D,E). Notably, strawberries with suppressed FxaC_21g15770 showed increased firmness with values of 16.22 N, representing a significant improvement of approximately 10% compared to the control group (Figure 8F). These results suggest that FxaC_21g15770 plays a pivotal role in strawberry fruit softening.

The cellulose and hemicellulose contents in strawberry fruits with a silenced FxaC_21g15770 gene were quantified as 36.15 mg/g and 0.41 mg/g, respectively, while those in strawberry fruits without the FxaC_21g15770 gene silencing were determined to be 40.83 mg/g and 0.39 mg/g, respectively (Figure 8G,H). No significant difference was observed in the cellulose and hemicellulose contents between the FxaC_21g15770-silenced and control groups. Furthermore, the soluble pectin content and original pectin content were measured at 0.27 mg/g and 0.66 mg/g, respectively, for strawberries with silenced FxaC_21g15770 gene expression, whereas they were found to be 0.78 mg/g and 0.35 mg/g in the control group (Figure 8I). Compared to the control group, there was a significant reduction of approximately 65% in soluble pectin content after silencing FxaC_21g15770 expression (Figure 8I). 

### 3.8. Effects of Transient Silencing of FxaC_21g15770 on Expression of Strawberry Ripening and Softening Related Genes

**Figure 9:**
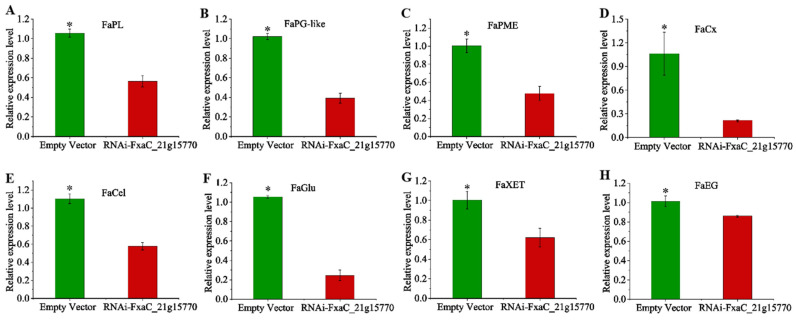
Effect of instantaneous silencing on the expression of ripening and softening related genes in strawberry. (**A**–**H**) Relative expression level of *FaPL*, *FaPG-like*, *FaPME*, *FaCx*, *FaCel*, *FaGlu*, *FaXET*, and *FaEG* in FxaC_21g15770 silenced fruits and control fruits. Single asterisk represent significant differences between the two samples at *p* ≤ 0.05 level.

The expression analysis of genes related to strawberry fruit ripening and softening showed that (Figure 9) the expressions of *FaPL*, *FaPG-like*, *FaPME*, *FaCx*, *FaCel*, *FaGlu*, *FaXET*, and *FaEG* were down-regulated in the silenced group compared with the control group, with the expressions down-regulated by 61%, 46%, 55%, 81%, 41%, 76%, 40%, and 20%, respectively. It indicated that the FxaC_21g15770 gene effectively inhibited the expression of genes related to cell wall decomposition to some extent, and increased the hardness of the strawberry.

## 4. Discussion

PG genes have been extensively studied in plant growth, development, fruit softening, and ripening. In the model plant, *Arabidopsis thaliana*, rice and tomato, 69, 46, and 45 family members have been identified [26,27,28], respectively. Similarly, in horticultural fruit crops, such as sweet cherry [29], kiwi [30], pear [31], peach [32], and grape [33], 45, 51, 61, 45, and 36 PG family members have been identified, respectively. These findings indicate the widespread distribution of the *PG* gene family across various plant species. Moreover, *FaPG1* and *FaPG2* genes have also been discovered in ‘Fragrance’ and ‘Sweet Charlie’ strawberry varieties [34]. However, a comprehensive analysis of the entire *PG* gene family in strawberry has not been published in the literature. In the present study, a total of 75 *FaPG* genes were identified in the ‘Benihoppe’ strawberry, which was increased compared to other mentioned species. This expansion is believed to be species-specific, and a result of gene tandem duplication events and chromosome replication during the evolution of the strawberry [35]. Furthermore, these 75 *FaPG* genes were widely distributed in 27 of the 28 cultivated strawberry chromosomes (Figure 1), suggesting that *FaPGs* may be involved in the regulation of multiple functions. Phylogenetic tree construction divided *FaPG* genes into three distinct groups (Figure 2), as opposed to the five groups in tomato [23] and peach [27]. Different gene groups exhibited unique motifs that support their close evolutionary relationship, and different motifs may play diverse roles in biological processes [36]. Notably, in this study, motif 2 was conserved among almost all *FaPG* genes while only four members lacked it, suggesting its relative conservation within the *FaPG* family. In addition, the gene structure of family members is closely related to their gene expression and functional variation [34]. Here, we found that the strawberry *FaPG* family exhibited certain regularities in terms of intron and exon distribution. For example, group c contained genes with more than 10 exons, while groups a and b predominantly had 4–7 exons (Figure 3). This observation indicates the highest level of intron/exon variation between groups and highlights the high degree of conservation within each group. A similar pattern of intron/exon structure has also been observed in the *MdPG* gene family of apple [37], where the number of exons varied from 1 to 18. Moreover, closely related genes such as FxaC_4g09610 and FxaC_4g12140 displayed comparable gene structures, exon lengths, and locations. In tomato, for instance, the *PG* gene (M37304) associated with fruit ripening was found to contain 8 introns, whereas the PG genes (AF000999, AF001001) linked to organ shedding had only 3 introns. This suggests that different degrees of intron insertion and deletion may indicate functional differentiation. Gene duplication plays an irreplaceable role in the expansion of gene families in the genome. In the genomes of rice and upland cotton, 10 and 129 fragment repeat gene pairs have been identified, respectively. In this study, a total of 118 pairs of genes were identified in the cultivated strawberry. Compared with rice, gene expansion was significant, which may be the result of tandem duplication. This is because all repeat events occurred on different chromatids of the same chromosome (Figure 4). 

Fruit softening serves as a critical indicator for assessing strawberry ripeness and plays an essential role in determining storage duration and overall quality impact. Excessive softening substantially reduces economic value [2,4]. Consequently, there is growing research interest in developing techniques that can effectively control fruit softening while preserving color, aroma, and nutritional content. Recent studies have revealed that PG is indeed one of the crucial genes involved in cell wall modification, with its expression found to be associated with fruit ripening across different species. For instance, during the fruit ripening process, five *SlPG* genes in tomatoes, two *PpPG* genes in peaches, and two *FaPG* genes in strawberries accumulate [26,32,34]. This accumulation enhances PG enzyme activity and subsequently promotes the cleavage of 1,4-2-D-galactose bonds, leading to glucuronate generation. Consequently, it triggers cell wall disintegration and accelerates pectin degradation. Conversely, when the *PG* gene is silenced or suppressed experimentally within fruits, cells become more compacted, resulting in stronger cell adhesion and increased fruit hardness. However, compared to other fruits, the detection of *PG* enzyme activity in strawberry is relatively low and presents challenges [38]. This observation suggests that this enzyme may play a secondary role in strawberry ripening. However, in this study, the transcription level of three *FaPG* members including FxaC_21g15770, FxaC_20g05360, and FxaC_19g05040 exhibited a gradual increase during fruit ripening and reached its peak in the FR stage of the low-hardness mutant. This finding strongly suggests that these three *FaPG* members may play crucial roles in strawberry fruit softening. Furthermore, FxaC_21g15770 was found to be the most-changed gene in expression, and was thereby selected for subsequent functional analysis. Functional validation of *PG* has been conducted in various fruits. Silencing *FaPG1* in strawberry [11] and *PpPG21/22* in peach [39] resulted in improved fruit hardness without affecting anthocyanin content or soluble solids levels. These results align with our result that silencing FxaC_21g15770 enhances strawberry fruit firmness, delays softening, and preserves coloration as well as soluble solids content (Figure 8F). Furthermore, we have aligned the gene sequence of FxaC_21g15770 to the *FaPG1* from ‘Chandler’ (accession: AF380299) and found that FxaC_21g15770 is a homologous gene of *FaPG1*. Therefore, our results confirming the important role of *FaPG1* in strawberry softening indicate that differential expression of FxaC_21g15770 might be the main reason for firmness differences between ‘Benihoppe’ and its mutant.

In addition, the PG gene primarily regulates pectin degradation. Silencing of such *PG* genes leads to an increase in protopectin content and a decrease in water-soluble pectin content and PG enzyme activity; however, it does not significantly affect cellulose and hemicellulose content [40]. Conversely, overexpression of raspberry *RiPG2* reduces fruit hardness while increasing water-soluble pectin content and PG enzyme activity; it also decreases protopectin, cellulose, and hemicellulose content [41]. These findings suggest that the impact of the *PG* gene on cellulose and hemicellulose content varies among different species. In this experiment, silencing FxaC_21g15770 significantly reduced soluble pectin content and PG activity while significantly increasing protopectin content; however, it had no effect on cellulose or hemicellulose levels (Figure 8G–I). It is possible that in addition to its structural role as a pectin depolymerase, *PG* also exerts regulatory effects on the expression of genes associated with cellulose and polysaccharides through other signaling pathways, thereby modulating the composition of the cell wall and facilitating fruit softening [9]. Genes such as *FaPL*, *FaPME*, *FaCX*, *FaCel*, *FaGlu*, *FaXET*, and *FaEG* are responsible for cellulose decomposition, pectin degradation, and polysaccharide breakdown during ripening processes leading to fruit softening [5,42,43]. In strawberry fruits specifically, *PL*, *PME*, and *EG*, which are involved in pectin decomposition mechanisms, promote the conversion of protopectin into soluble pectin, thus mediating fruit softening [44,45,46]. Following fruit ripening stages, there is a rapid increase in the expression levels of cell wall metabolism-related genes. Studies have demonstrated that overexpression of the raspberry *RiPG2* gene can enhance PG enzyme activity to facilitate pectin degradation while positively regulating the expression of *PME* and *PL* genes. At the same time, it may also regulate the expression of enzymes involved in cellulose and hemicellulose degradation, such as CX, Cel, Glu, XET, and EG, through other positive feedback signals. This regulation can subsequently alter the composition of the cell wall and facilitate fruit softening [41]. It is evident that silencing the PG gene should result in the down-regulation of related enzyme genes. In this experiment, a significant down-regulation was observed in the expression levels of *FaPG-like*, *FaPL*, *FaPME*, *FaCX*, *FaCel*, *FaGlu*, *FaXET*, and *FaEG* when compared to the control (Figure 9), which aligns with previous research findings. The inhibition of protopectin decomposition by FxaC_21g15770 could potentially increase strawberry hardness by suppressing the expression of key genes including *FaPG-like, FaPL*, *FaPME*, and *FaEG*. The down-regulation of enzymes associated with cellulose and hemicellulose degradation, namely *FaCX*, *FaCel*, *FaGlu*, *FaXET*, and *FaEG*, without significant changes in the content of cellulose and hemicellulose may suggest that they do not play a crucial role in the ripening and softening processes of strawberry fruits [47,48]. Instead, their main impact lies in altering the viscosity and porosity of the cell wall while inhibiting xyloglucan degradation. This effectively suppresses PG enzyme activity and subsequently impedes softening. However, we did not investigate the expression of other *FaPG* members in the FxaC_21g15770-silenced sample, and whether there is a compensation mechanism among *FaPGs* when one of the members is silenced needs further research.

## 5. Conclusions

To sum up, in this study encompassing analysis of the ‘Benihoppe’ strawberry cultivar alongside its softer mutant, we identified 75 *FaPG* genes within the octoploid strawberry genome. We further investigated their physical and chemical properties as well as their chromosomal location through phylogenetic development analysis along with gene structure examination. Through these efforts combined with our findings from mutant analysis, we successfully pinpointed three *FaPG* members potentially associated with strawberry ripening and softening. Among them, a most-changed gene named FxaC_21g15770 (homologous with *FaPG1*) could negatively regulate strawberry fruit hardness without affecting fruit color, soluble solids, cellulose, and hemicellulose. Moreover, silencing of FxaC_21g15770 could significantly suppress the other genes related to pectin degradation, such as *FaPG-like*, *FaP*L, *FaPME*, *FaCX*, *FaCel*, *FaGlu*, *FaXET*, and *FaEG*. Our results provided basic information regarding the FaPG gene family, and identified the potential members crucial for strawberry softening, thereby offering new insights towards unraveling the underlying mechanisms governing strawberry fruit softening.

## Data Availability

All relevant data are available within the article and its supplementary data. The data presented in the study are deposited in the NCBI SRA database, accession number PRJNA838938.

## Supplementary Materials

The following supporting information can be downloaded at https://www.mdpi.com/article/10.3390/plants13131838/s1. Table S1: *FaPG1* Primers for amplification of gene silencing vector construction. Table S2: Primers used for the RT-qPCR in this study. Table S3: Physicochemical characteristics of *FaPG* proteins.
