# Supplementary material for: Genome-Wide Analysis of Polygalacturonase Gene Family Reveals Its Role in Strawberry Softening
Source: Plants (Basel). 2024 Jul 4;13(13):1838. doi: 10.3390/plants13131838 (PMC11244104; doi:10.3390/plants13131838)
Supplement: Supplementary file 1 [file plants-13-01838-s001.zip › plants-3029954-supplementary.pdf]

Supplementary Material

Genome-wide analysis of polygalacturonase gene family reveals its role in strawberry softening

1 Supplementar Tables

Table S1 *FaPG1* Primers for amplification of gene silencing vector construction

Target gene	Primer sequences（5’-3’）
FaPG1	*RNAi-FaPG1-A*： GGAATGTAAGATGTTCACACCC
	RNAi-FaPG1-B： GCAGCAACAACGCCAAGTAG
	RNAi-FaPG1-C: GCAGCAACAACGCCAAGTAGAGT
	RNAi-FaPG1-D： GGAATGTAAGATGTTCACACCC

Table S2 Primers used for the RT-qPCR in this study

Target gene	Primer sequences（5’-3’）
FaActin-F:	TGGGTTTGCTGGAGATGAT
FaActin-R:	X1CAGTTAGGAGAACTGGGTGC
FaPG1-F:	CAACCGGGATTAACATTACGGAC
FaPG1-R:	CTTCATTGTCGTATCTTCCGAGAC
FaPG-like-F:	TGTTGCTAACCCAGTCCTCAT
FaPG-like-R:	CGCAGTTGAAGTTGTCCCTAT
FaPL-F:	GACTCCCTTGCTGCTTCTTTT
FaPL-R:	CGTGGAACTGTTACGAATGCT
FaPME-F:	TGGTGCTGGTTTTCTTATCGTT
FaPME-R:	CGGAGAGAATAGACACCACCTG
FaCX-F:	TGTGTGTCCGTTCTATTGTGA
FaCX-R:	AGATTGAATGTAGCCCAGGTA
FaCel-F:	GCTCTGTTTTGCCTGGACTT
FaCel-R:	GCGTGGCTTAGATAGTTGGAAT
FaXET-F:	CTTCCACTCCTACTCCATTTTC
FaXET-R:	CAACCCCTTGCTTTCCAT

FaEG-F:	TGTAACTTCGCTATCGTTCCTG
FaEG-R:	TGTCACCCAAAATGTAATCCAC
FaGlu-F:	GGCAAACTCAAATACCACAA
FaGlu-R:	CCCTCTTACATCTGCTCCTT

Table S3 Physicochemical characteristics of *FaPG* proteins

Gene ID	Amino acid/aa	ORF/bp	Molecular weight/ku	PI	Instablility index	Aliphatic index	GRAVY	Chromosome	Location	Subcellar localization
FxaC_15g03310	603	1809	65588.84	9.23	44.23	75.47	-0.34	Fvb4-2	1575502 1580135	Vacuolar
FxaC_15g08790	154	462	16850.93	6.04	30.97	75.26	-0.399	Fvb4-2	4074141 4074690	Vacuolar
FxaC_7g01330	407	1221	44181.09	8.77	34.17	84.05	-0.186	Fvb2-1	789276 790614	Chloroplast
FxaC_7g16880	355	1065	39104.67	8.32	31.64	79.86	-0.229	Fvb2-1	11385822 11387640	Cytoplasmic
FxaC_7g26380	441	1323	48082.81	5.58	42.69	77.1	-0.163	Fvb2-1	16943230 16945749	Nuclear
FxaC_12g49110	297	891	32268.94	9.38	23.85	83.37	-0.018	Fvb3-1	31191868 31194096	Nuclear
FxaC_21g14210	357	1071	38975.84	6.19	35.09	84.06	-0.193	Fvb6-1	6335043 6336116	Cytoplasmic
FxaC_21g15770	405	1215	43228.21	8.33	32.44	84.47	-0.143	Fvb6-1	7054540 7056595	Chloroplast stroma
FxaC_21g15750	412	1236	43677.59	6.59	29.72	80.97	-0.208	Fvb6-1	7048185 7050193	Chloroplast
FxaC_21g15780	398	1194	42657.03	8.49	30.14	80.53	-0.254	Fvb6-1	7060071 7071469	Vacuolar
FxaC_18g41830	440	1320	48179.79	8.63	39.45	91.25	-0.061	Fvb5-3	25115592 25120700	Vacuolar
FxaC_25g04180	469	1407	51179.88	9.64	34.19	84.61	0.264	Fvb7-2	2696731 2699743	Nuclear
FxaC_25g06550	414	1242	45092.02	5.99	29.05	89.47	-0.147	Fvb7-2	4103127 4109904	Nuclear
FxaC_11g15570	423	1269	46719.85	9.22	30.47	90.33	-0.028	Fvb3-3	7879132 7881306	Cell membrane
FxaC_11g23660	233	699	24839.76	7.67	53.41	77.38	-0.39	Fvb3-3	13061667 13062477	Cell membrane
FxaC_4g01710	364	1092	39973.15	6.66	42.45	83.57	-0.326	Fvb1-1	1567116 1568717	Cell membrane
FxaC_4g07280	394	1182	42901.71	7.09	37.63	83.12	-0.198	Fvb1-1	7505976 7507626	Cell membrane.
FxaC_4g09430	466	1398	51296.83	5.69	43.86	89.27	-0.127	Fvb1-1	9327437 9331494	Cell membrane
FxaC_4g09610	142	426	15810.99	8.65	53.17	97.39	-0.194	Fvb1-1	9483675 9484547	Cell membrane.
FxaC_4g12140	475	1425	51957.15	6.2	44.15	84.15	-0.135	Fvb1-1	11774604 11779518	Cytoplasmic
FxaC_4g12250	475	1425	51957.5	6.2	44.15	84.15	-0.135	Fvb1-1	11836053 11840988	Cytoplasmic
FxaC_4g15600	365	1095	38451.42	8.22	27.42	78.22	-0.093	Fvb1-1	14411403 14413712	Chloroplast stroma
FxaC_4g31340	506	1518	57137.41	8.64	43.08	89.33	-0.219	Fvb1-1	23526095 23531153	Chloroplast stroma
FxaC_9g06620	463	1389	49473.91	4.92	37.72	90.26	-0.044	Fvb3-4	3457860 3459295	Chloroplast stroma
FxaC_16g07090	235	705	25387.72	5.81	49.68	83.36	-0.051	Fvb4-1	4992739 4994985	Chloroplast stroma
FxaC_16g23340	579	1737	63010.9	8.94	41.02	78.24	-0.276	Fvb4-1	16214813 16215091	Nuclear
FxaC_16g28810	579	1737	63010.9	8.94	41.02	78.24	-0.276	Fvb4-2	16288231 16293708	Chloroplast stroma
FxaC_22g13530	412	1236	43701.6	6.32	27.15	81.17	-0.219	Fvb6-3	6505318 6507083	Vacuolar
FxaC_22g13540	434	1302	46561.52	6.88	34.94	85.53	-0.194	Fvb6-3	6508377 6513115	Cytoplasmic
FxaC_22g13810	393	1179	41655.97	6.1	27.44	76.67	0.307	Fvb6-3	6679779 6681539	Cytoplasmic
FxaC_23g58370	412	1236	43685.6	6.32	27.62	81.65	-0.212	Fvb6-2	34262998 34264920	Vacuolar
FxaC_22g13830	1513	4539	160514.19	5.91	27.04	77.65	-0.279	Fvb6-3	6708009 6732994	Chloroplast
FxaC_22g17550	389	1167	42400.04	8.4	33.18	82.96	-0.136	Fvb6-3	8714535 8715704	Vacuolar
FxaC_1g04650	551	1653	62077.51	5.53	33.93	85.57	-0.23	Fvb1-4	1945037 1950157	Vacuolar
FxaC_1g14020	848	2544	90853.97	6.23	34.38	88.88	-0.066	Fvb1-4	6079102 6084713	Vacuolar

FxaC_1g29290	481	1443	52808.19	8	46.51	83.49	-0.178	Fvb1-4	14527366	14532526	Cytoplasmic
FxaC_1g32280	457	1371	50278.63	5.51	41.25	89.74	-0.109	Fvb1-4	16557240	16562491	Vacuolar
FxaC_1g33520	351	1053	38158.31	8.61	40.55	75.58	-0.373	Fvb1-4	17346576	17348684	Nuclear
FxaC_1g33960	426	1278	46448.58	8.95	34.89	77.58	-0.368	Fvb1-4	17560439	17562455	Nuclear.
FxaC_23g08920	492	1476	53892.76	8.48	39.54	93.9	0.051	Fvb6-2	5586378	5589903	Vacuolar
FxaC_23g58410	288	864	31132.72	5.29	41.45	89.34	-0.002	Fvb6-2	34271897	34273165	Cytoplasmic
FxaC_17g03410	443	1329	48399.98	8.62	41.02	91.06	-0.089	Fvb5-1	1680134	1683595	Cytoplasmic
FxaC_17g05730	443	1329	48397.96	8.62	40.4	90.18	-0.101	Fvb5-1	2844163	2849372	Chloroplast
FxaC_17g06680	207	621	22065.08	7.06	34.77	92.71	0.067	Fvb5-1	3241463	3246701	Chloroplast
FxaC_3g03490	476	1428	52253.51	7.1	41.43	82.94	-0.173	Fvb1-3	1598727	1599742	Cytoplasmic
FxaC_3g29420	466	1398	51301.85	5.74	45.14	89.06	-0.13	Fvb1-3	17189409	17196647	Chloroplast
FxaC_3g32660	322	966	35830.81	8.83	51.36	76.24	-0.244	Fvb1-3	19549504	19554088	Nuclear
FxaC_3g33100	344	1032	37734.96	7.14	43.83	86.16	-0.294	Fvb1-3	19841705	19843444	Vacuolar
FxaC_3g33740	416	1248	44202.06	6.1	30.5	80.87	-0.244	Fvb1-3	6847146	6849465	Cytoplasmic
FxaC_24g50180	470	1410	51426.96	5.9	42.61	82.68	-0.076	Fvb6-4	30272424	30274450	Vacuolar
FxaC_8g02340	499	1497	54369.45	8.42	33.06	80.26	-0.22	Fvb2-3	1439819	1442405	Vacuolar
FxaC_8g37960	921	2763	100557.45	8.96	41.72	76.73	-0.325	Fvb2-3	22230765	22232371	Vacuolar
FxaC_13g03530	117	351	12949.25	8.93	23.08	104.1	0.33	Fvb4-3	1551092	1558405	Vacuolar
FxaC_13g27210	484	1452	52671.68	7.66	37.83	93.68	0.024	Fvb4-3	13262916	13263615	Chloroplast
FxaC_13g31420	117	351	12963.27	8.93	23.08	104.96	0.326	Fvb4-3	15741815	15743992	Cytoplasmic
FxaC_13g37980	491	1473	55205.26	9.11	42.82	89.29	-0.199	Fvb4-3	19897028	19897727	Nuclear
FxaC_2g00610	461	1383	50811.94	8.85	44.33	91.95	-0.111	Fvb1-2	344111	348460	Chloroplast stroma
FxaC_2g08520	491	1473	55202.26	9.11	42.82	89.29	-0.199	Fvb1-2	3897760	3901029	Vacuolar
FxaC_2g14070	491	1473	55202.26	9.11	42.82	89.29	-0.199	Fvb1-2	6249292	6253641	Chloroplast stroma
FxaC_2g30590	277	831	29119.21	8.95	28.58	94.26	0.133	Fvb1-2	15660486	15661642	Cytoplasmic
FxaC_19g03010	530	1590	58227.31	6.03	41.47	82.21	-0.132	Fvb5-4	1556617	1560502	Vacuolar
FxaC_19g05040	443	1329	48442.06	8.63	39.57	91.06	-0.064	Fvb5-4	2512094	2517341	Chloroplast
FxaC_6g01680	577	1731	63405.27	9.18	34.6	85.3	-0.204	Fvb2-4	983685	985525	Chloroplast
FxaC_6g20540	420	1260	45492.1	9.33	32.21	77.52	-0.123	Fvb2-4	13158298	13167209	Cell membrane
FxaC_6g25060	450	1350	49638.35	8.69	35.62	70.18	-0.429	Fvb2-4	15858095	15860388	Vacuolar
FxaC_6g36110	450	1350	49638.35	8.69	35.62	70.18	-0.429	Fvb2-4	21682866	21685159	Vacuolar
FxaC_5g09420	513	1539	56468.14	6.3	40.93	86.78	0.047	Fvb2-2	4385708	4388499	Chloroplast stroma
FxaC_5g20690	747	2241	81701.48	7.74	34.19	89.29	-0.127	Fvb2-2	9881805	9887291	Vacuolar
FxaC_5g41630	498	1494	54329.42	8.56	33.57	79.84	-0.226	Fvb2-2	21942321	21943929	Vacuolar
FxaC_10g13440	1053	3159	117498.18	6.21	46.08	83.06	-0.283	Fvb3-2	6572506	6576924	Cell membrane
FxaC_14g03510	436	1308	47579.47	6.31	35.73	92.71	0.008	Fvb4-4	1694695	1697637	Cytoplasmic
FxaC_26g38720	405	1215	43591.06	9.26	27.03	82.81	-0.171	Fvb7-3	21885119	21887086	Chloroplast
FxaC_28g11240	125	375	13791	9.26	37.15	84.96	-0.263	Fvb7-4	5365443	5365905	Nuclear
FxaC_28g34160	460	1380	50915.31	5.95	30.22	78.8	-0.265	Fvb7-4	18582579	18585052	Endoplasmic reticulum
FxaC_20g05360	443	1329	48286.91	8.63	39.21	92.6	-0.053	Fvb5-2	2748027	2753075	Chloroplast
